# Clinico-pathology, hematology, and biochemistry responses toward *Pasteurella multocida* Type B: 2 via oral and subcutaneous route of infections

**DOI:** 10.14202/vetworld.2015.783-792

**Published:** 2015-06-24

**Authors:** Eric Lim Teik Chung, Faez Firdaus Jesse Abdullah, Lawan Adamu, Ali Dhiaa Marza, Hayder Hamzah Ibrahim, Mohd Zamri-Saad, Abdul Wahid Haron, Abdul Aziz Saharee, Mohd Azmi Mohd Lila, Abdul Rahman Omar, Md Zuki Abu Bakar, Mohd Jefri Norsidin

**Affiliations:** 1 Department of Veterinary Clinical Studies, Faculty of Veterinary Medicine, Universiti Putra Malaysia, 43400 Serdang, Selangor, Malaysia; 2 Department of Ruminant Disease, Research Centre for Ruminant Disease, Universiti Putra Malaysia, 43400 Serdang, Selangor, Malaysia; 3 Department of Veterinary Medicine, Faculty of Veterinary Medicine, University of Maiduguri, PMB1069, Borno State, Nigeria; 4 Department of Veterinary Internal Medicine, Faculty of Veterinary Medicine, Al-Qasim Green University, Iraq; 5 Department of Veterinary Medicine, Technical Institute Babil, Al Furat Alawast Technical University, Iraq; 6 Department of Veterinary Pathology and Microbiology, Faculty of Veterinary Medicine, Universiti Putra Malaysia, 43400 Serdang, Selangor, Malaysia; 7 Department of Preclinical, Faculty of Veterinary Medicine, Universiti Putra Malaysia, 43400 Serdang, Selangor, Malaysia

**Keywords:** buffalo heifers, clinico-pathology, hematology and biochemistry responses, oral route, *Pasteurella multocida* Type B:2, subcutaneous route

## Abstract

**Background:**

*Pasteurella multocida* a Gram-negative bacterium has been identified as the causative agent of many economically important diseases in a wide range of hosts. Hemorrhagic septicemia is a disease caused by *P. multocida* serotype B:2 and E:2. The organism causes acute, a highly fatal septicemic disease with high morbidity and mortality in cattle and more susceptible in buffaloes. Therefore, the aim of this study was to investigate the clinical signs, blood parameters, post mortem and histopathology changes caused by *P. multocida* Type B:2 infections initiated through the oral and subcutaneous routes.

**Methods:**

Nine buffalo heifers were divided equally into 3 treatment groups. Group 1 was inoculated orally with 10 ml of phosphate buffer saline; Groups 2 and 3 were inoculated with 10 ml of 10^12^ colony forming unit of *P. multocida* Type B:2 subcutaneously and orally respectively.

**Results:**

There was a significant difference (p<0.05) in temperature between the subcutaneous and the control group. The results revealed significant differences (p<0.05) in erythrocytes, hemoglobin, packed cell volume, leukocytes, monocytes, and A: G ratio between the subcutaneous and the control group. Furthermore, there were significant differences (p<0.05) in leukocytes, band neutrophils, segmented neutrophils, lymphocytes, eosinophils, basophils, thrombocytes, plasma protein, icterus index, gamma glutamyl tranferase and A: G ratio between the oral and the control group. The post mortem lesions of the subcutaneous group buffaloes showed generalized hyperemia, congestion and hemorrhage of the immune organs, gastro-intestinal tract organs and vital organs. The oral group buffaloes showed mild lesions in the lung and liver. Histologically, there were significant differences (p<0.05) in hemorrhage and congestion; necrosis and degeneration; inflammatory cells infiltration; and edema in between the groups.

**Conclusion:**

This study was a proof that oral route infection of *P. multocida* Type B:2 can be used to stimulate host cell responses where oral vaccine through feed can be developed in the near future.

## Introduction

Hemorrhagic septicemia (HS) disease is a specific form of Pasteurellosis in cattle and buffalo which is different from other Pasteurellosis that play only a secondary role [1]. HS is an acute, fatal, and septicemic disease of cattle and buffaloes caused by a specific serotype of *Pasteurella multocida* which is a Gram-negative coccobacillus [2-4]. Using a combination of capsular and somatic typing, the two common HS serotypes popularly known as the Asian and African serotypes are designated B:2 and E:2 respectively [5,6]. Buffaloes are more susceptible to the disease and usually occur more frequently in poor husbandry conditions and in countries with disease surveillance that is not well developed [2,7,8]. The disease is of great economic importance in Malaysia and throughout South-east Asia where cattle and buffaloes are abundant for beef and milk production [9-13].

Sudden death in HS is usually the first report among free-ranging animals during outbreaks. There are four clinical syndromes in a diseased animal. Animal will first exhibit elevated temperature above 40°C, followed by submandibular edema and then respiratory distress with profuse nasal discharge, and finally recumbency and death [2]. At post mortem, the most obvious lesions in affected animals are the edema, widely distributed hemorrhages, and generalized hyperemia. In most cases, there will be also clear or straw colored edematous fluid at the head, neck, brisket and musculature region. Petechial hemorrhages are particularly prominent in the pharyngeal and cervical lymph nodes. Besides that, blood tinge fluid is often found in the pericardial sac, thoracic and abdominal cavity [11,14,15]. Meningitis was also observed [16]. Histopathological lesions such as hemorrhage, hyperemia, edema and white blood cells infiltration were observed in the lung, lymph nodes, spleen, gastro-intestinal tract, liver, kidney and the heart [17]. Nevertheless, knowledge on the changes in the immune system organs had yet to be uncovered. Information on this will play a significant role in understanding the pathogenesis of *P. multocida* Type B:2.

There are still many grey areas in the knowledge of HS. Significant gaps exist in understanding the pathogenesis of the disease [2]. The aim of this study was to investigate the clinical responses, hematology and biochemistry alterations, post mortem changes, and cellular changes in tissues of buffaloes challenged with *P. multocida* Type B:2 via oral and subcutaneous routes.

## Materials and Methods

### Ethical approval

This research was approved by the Animal Care and Use Committee of Universiti Putra Malaysia (approval number: R056/2014).

### Animal selection

A 8-month-old, clinically healthy, non-pregnant and non-lactating buffalo heifers were used in this study. On arrival at the Animal Experimental House, Faculty of Veterinary Medicine, Universiti Putra Malaysia, 1 ml/50 kg of anthelmintic (Ivermectin) was administered subcutaneously to control internal parasitism, which has been shown to influence disease development [15]. Besides that, nasal swabs were also collected from all buffaloes to ensure that the animals were free from *P. multocida* prior to the start of the experiment. The buffaloes were placed in an individual pen and were fed with cut grass and supplement with pellets at the rate of 1 kg/animal/day. Water was available ad libitum.

### Inoculums preparation

Wild-type *P. multocida* used in this study was isolated from a previous outbreak of HS in the state of Kelantan, Malaysia. The isolate was confirmed to be *P. multocida* Type B:2 via Gram-staining method, biochemical test and polymerase chain reaction method. Bacteria were then cultured on blood agar plates and incubated at 37°C for 24 h before the concentration was determined by McFarland Nephelometer Barium Sulfate Standards.

### Experimental design

All the 9 buffalo heifers were divided equally into 3 treatment groups. Group 1 was the negative control group where the buffaloes were inoculated orally with 10 ml of phosphate buffer saline. Group 2 was the positive control group and was inoculated subcutaneously with 10 ml of 10^12^ colony forming unit (CFU) of *P. multocida* Type B:2. Group 3 was the treatment group where the buffaloes were inoculated orally with 10 ml of 10^12^ CFU of *P. multocida* Type B:2 using a stomach tube. During the post-infection period, all the buffaloes were observed for clinical signs and clinical response throughout 21 days. The clinical signs that were observed include temperature, heart rate, respiratory rate, mucous membrane, rumen motility, salivation, nasal discharges, edema swelling, movement, and dullness. Blood samples were collected at a predetermined interval. The blood samples were collected via jugular venipuncture into 5 ml plain vacutainers and ethylenediaminetetraacetic acid tubes for complete blood count and biochemistry analyses. At the end of the study, surviving buffaloes after 21 days were euthanized for post-mortem evaluation. Immune organs, gastro-intestinal tract organs and vital organs samples were collected for microscopic examinations and cellular changes evaluation. The immune organs consist of bone marrow, spleen, submandibular lymph nodes, prescapular lymph nodes, femoral lymph nodes, mesenteric lymph nodes and tonsil. The gastrointestinal tract organs collected were esophagus, rumen, reticulum, omasum, abomasum, duodenum, jejunum, ileum, caecum, colon, and rectum. The vital organs collected were lung, heart, liver, and kidneys.

### Histopathology analysis and lesion scoring

The samples were preserved in 10% formalin before they were processed using routine histology slide preparation technique and stained with hematoxylin and eosin stain. The cellular changes observed were hemorrhage and congestion; necrosis and degeneration; inflammatory cells infiltration; and edema lesion. These cellular changes were then scored into 4 scores, which include score 0: Normal (normal tissue); score 1: Mild (<25% tissue affected); score 2: Moderate (<50% tissue affected); and score 3: Severe (more than 50% tissue affected).

### Statistical analysis

All the data were analyzed using JMP^®^ 11. NC: SAS Institute Inc. software Version. The data were considered significant at p<0.05.

## Results

### Clinical response

**Figure-1:**
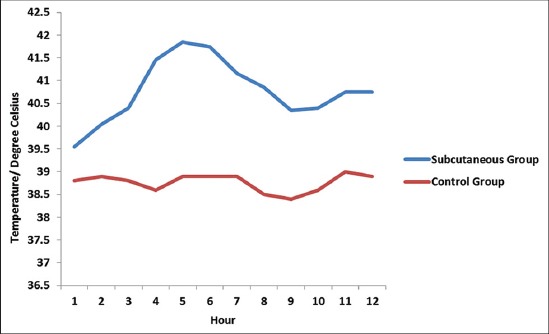
Mean rectal temperatures for the subcutaneous group after 12 h of inoculation with *Pasteurella multocida* Type B:2.

**Figure-2:**
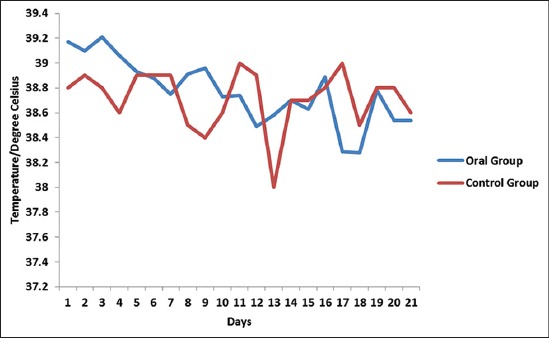
Mean rectal temperatures for the oral group after 21 days of inoculation with *Pasteurella multocida* Type B:2.

Buffaloes from Group 1 showed normal clinical finding throughout 21 days. The temperature, heart rate, respiratory rate, mucous membrane, and rumen motility were within the normal range. There were no salivation, nasal discharges, edema swelling, and dullness observed. On the other hand, buffaloes from Group 2 showed typical HS clinical signs and were only able to survive for the first 12 h of the experiment. At 3 h post-infection, all buffaloes started to have serous nasal discharge followed by congested mucous membrane after 4 h, submandibular edema and dullness after 5 h, and finally mucopurulent nasal discharge with respiratory distress and absent of rumen motility after 11 h post infection. All buffaloes from Group 2 were euthanized at 12 h post infection following the Animal Welfare Guidelines where the animals were in recumbency and were having respiratory distress. Group 2 rectal temperatures were high throughout the experimental period where the temperatures were above 39°C (Figure-1). In contrast, Group 3 buffaloes were able to survive throughout the 21 days experiment regardless of showing some mild clinical response. High rectal temperatures (Figure-2) and serous nasal discharges were only observed for the first 4 days. The rumen motility was normal and was not affected by oral inoculation of *P. multocida* Type B:2. After day 5, all parameters were within the normal range. There were significant differences (p<0.05) in temperature between the subcutaneous and oral group compared to the control group.

### Hematology and biochemistry

From the hematology and biochemistry results, there were no significant findings observed in Group 1 buffaloes. However, Group 2 buffaloes showed erythrocytosis, leukopenia and lymphopenia throughout the 12 h of the experiment. In Group 3, all buffaloes were only having leukocytosis for the first 5 days. Other parameters were within the normal range. There were significant differences (p<0.05) in erythrocytes, hemoglobin, packed cell volume (PCV), leukocytes, monocytes, and A: G ratio between the subcutaneous and control group. However, there were no significant differences (p>0.05) in the other parameters (Table-1). In contrast, the oral and control group revealed significant differences (p<0.05) in leukocytes, band neutrophils, segmented neutrophils, lymphocytes, eosinophils, basophils, thrombocytes, plasma protein, icterus index, gamma glutamyl transferase and A: G ratio. There were no significant differences (p>0.05) in the other parameters in buffaloes inoculated orally (Table-2).

**Table-1:** Haematological and biochemical alterations in buffaloes after 12 h of subcutaneous inoculation of *Pasteurella multocida* Type B:2.

Parameters	Control group	Subcutaneous group
Erythrocytes (×10^12^/L)	6.86±0.10_b_	8.66±0.20_a_
Haemoglobin (g/L)	120±1.78_b_	152.50±4.03_a_
PCV (L/L)	0.36±0.01_b_	0.44±0.01_a_
MCV (fL)	53±0.46_a_	50.56±0.26_a_
MCHC (g/L)	333±2.13_a_	347.83±2.94_a_
Leukocytes (×10^9^/L)	8±0.37_a_	3.72±0.99_b_
Band neutrophils (×10^9^/L)	0.10±0.02_b_	0.09±0.04_b_
Seg neutrophils (×10^9^/L)	2±0.3_b_	2.33±0.74_b_
Lymphocytes (×10^9^/L)	3.04±0.13_b_	1.08±0.25_b_
Monocytes (×10^9^/L)	0.66±0.03_a_	0.23±0.08_b_
Eosinophils (×10^9^/L)	0.00±0.00_b_	0.00±0.00_b_
Basophils (×10^9^/L)	0.00±0.00_b_	0.00±0.00_b_
Thrombocytes (×10^9^/L)	350±16.61_b_	299.89±35.77_b_
Plasma protein (g/L)	72±0.47_b_	65.89±0.99_b_
Icterus index (Unit)	2±0.18_b_	4.14±0.39_a_
GGT (U/L)	4±0.28_b_	3.00±0.28_b_
Total protein (g/L)	67.05±0.52_a_	63.49±0.55_a_
Albumin (g/L)	33.60±0.28_a_	32.99±0.47_a_
Globulin (g/L)	32.60±0.50_a_	30.50±0.33_a_
A:G (unit)	1±0.03_b_	1.08±0.02_a_

All values are expressed as mean±SE;

a,b values with superscript within rows are significantly different at p<0.05;

PCV=Packed cell volume, MCV=Mean corpuscular volume; MCHC=Mean corpuscular haemoglobin concentration, GGT=Gamma glutamyl tranferase; A:G=Albumin:Globulin ratio, SE=Standard error

**Table-2:** Haematological and biochemical alterations in buffaloes after 21 days of oral inoculation of *Pasteurella multocida* Type B: 2.

Parameters	Control group	Oral group
Erythrocytes (×10^12^/L)	6.86±0.10_b_	6.75±0.21_b_
Haemoglobin (g/L)	120±1.78_b_	120.32±3.17_b_
PCV (L/L)	0.36±0.01_b_	0.34±0.01_b_
MCV (fL)	53±0.46_a_	50.75±1.23_a_
MCHC (g/L)	333±2.13_a_	359.75±5.13_a_
Leukocytes (×10^9^/L)	8±0.37_b_	13.97±0.49_a_
Band neutrophils (×10^9^/L)	0.10±0.02_b_	0.25±0.02_a_
Seg neutrophils (×10^9^/L)	2±0.3_b_	5.30±0.48_a_
Lymphocytes (×10^9^/L)	3.04±0.13_b_	6.74±0.25_a_
Monocytes (×10^9^/L)	0.66±0.03_a_	0.79±0.05_a_
Eosinophils (×10^9^/L)	0.00±0.00_b_	0.72±0.13_a_
Basophils (×10^9^/L)	0.00±0.00_b_	0.18±0.04_a_
Thrombocytes (×10^9^/L)	350±16.61_b_	453.53±31.35_a_
Plasma protein (g/L)	72±0.47_b_	75.85±0.90_a_
Icterus index (Unit)	2±0.18_b_	3.95±0.33_a_
GGT (U/L)	4±0.28_b_	8.90±0.71_a_
Total protein (g/L)	67.05±0.52_a_	65.01±1.28_a_
Albumin (g/L)	33.60±0.28_a_	33.39±0.63_a_
Globulin (g/L)	32.60±0.50_a_	31.62±1.33_a_
A:G (Unit)	1±0.03_b_	1.13±0.06_a_

All values are expressed as mean±SE;

a,b values with superscript within rows are significantly different at *P*<0.05;

PCV=Packed cell volume, MCV=Mean corpuscular volume, MCHC=Mean corpuscular haemoglobin concentration, GGT=Gamma glutamyl tranferase, A:G=Albumin:Globulin ratio, SE=Standard error

### Gross lesions

**Figure-3:**
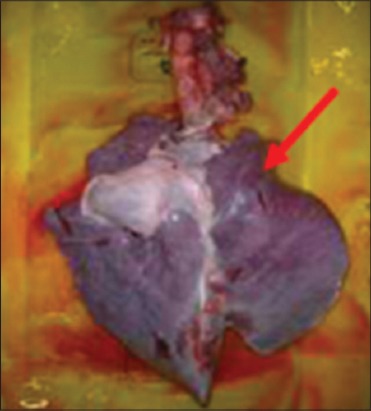
Congested and hemorrhagic lung.

**Figure-4:**
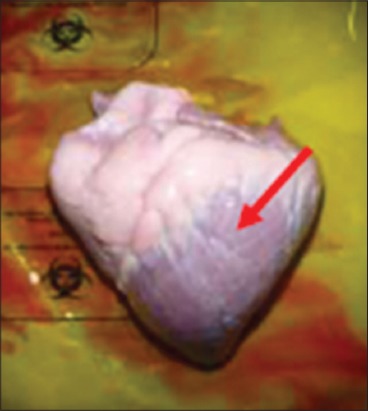
Congested and hemorrhagic heart.

**Figure-5:**
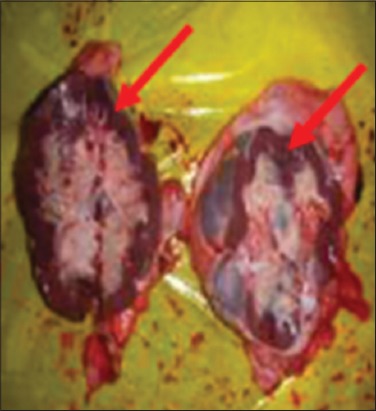
Congested and hemorrhagic kidneys.

**Figure-6:**
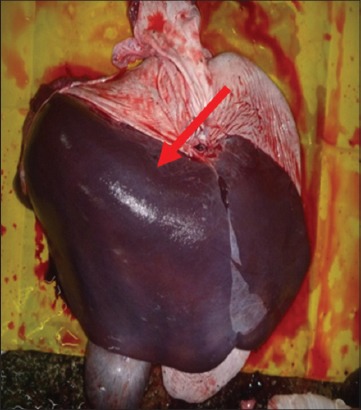
Congested and hemorrhagic liver.

**Figure-7:**
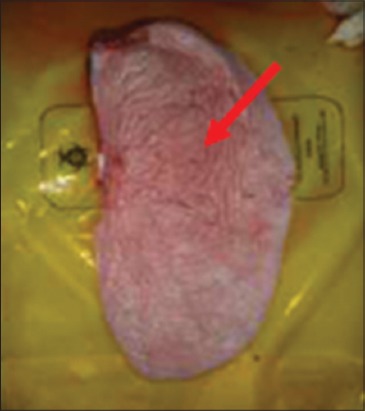
Congested and hemorrhagic spleen.

**Figure-8:**
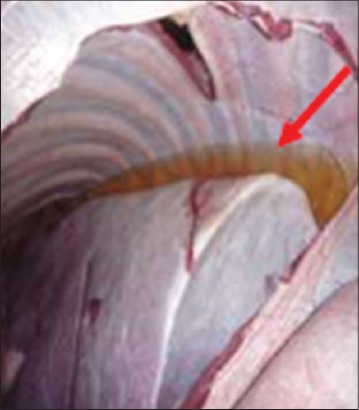
Straw colour fluid in thoracic region.

Buffaloes from Group 2 were euthanized after 12 h post infection due to recumbency and sign of respiratory distress. All vital organs including the lung (Figure-3), heart (Figure-4), kidney (Figure-5), liver (Figure-6) and spleen (Figure-7) appeared to be hyperemic, congested and hemorrhagic. The gastrointestinal tract such as esophagus, abomasum, duodenum, jejunum, ileum, caecum and rectum also showed similar findings. Straw colored blood tinge fluid was found in the thoracic region (Figure-8).

**Figure-9:**
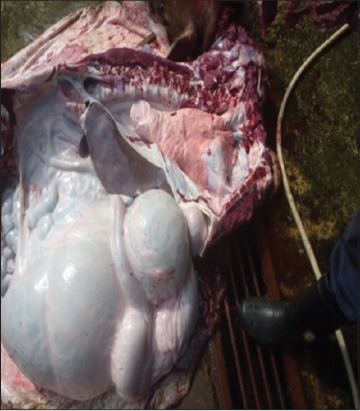
Normal organ *in-situ*.

**Figure-10:**
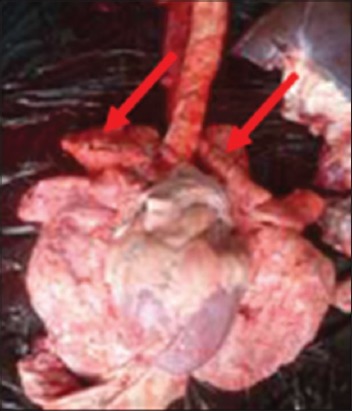
Congested and fibrinous cranial lobes.

**Figure-11:**
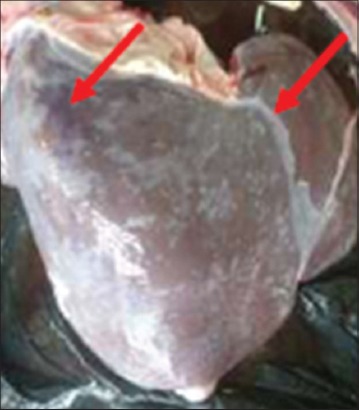
Fibrinous and hemorrhagic liver.

In contrast, Groups 1 and 3 buffaloes were euthanized only after 21 days for post mortem examination and organs sample collection. All the organs from Group 1 buffaloes appeared to be normal with no significant findings (Figure-9). For Group 3 buffaloes, there were gross lesions in the lung and liver. The left and right cranial lobes of the lung appeared to be congested, fibrinous and firm in consistency (Figure-10). It was consolidated with mosaic/marbling like appearance. Blood was oozing out upon cutting surface. This condition was also known as fibrinous pleuropneumonia. Besides that, the liver was having mild multifocal hemorrhage and fibrin deposition around the liver surface (Figure-11). Upon cutting the surface, blood was oozing out from the liver. Nevertheless, the immune organs, gastrointestinal organs, and other vital organs from Group 3 buffaloes appeared to be normal grossly.

### Histopathology

**Figure-12:**
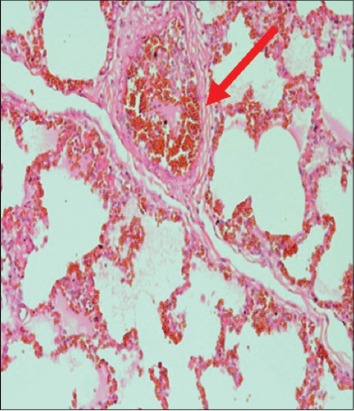
Micrograph of congestion and haemorrhagic in the lung, H and E, ×200, (Group 2).

**Figure-13:**
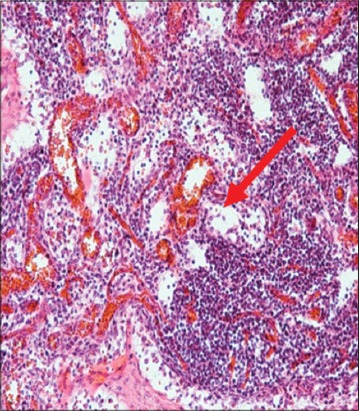
Micrograph of necrosis and degeneration in the submandibular lymph node, H and E, ×200, (Group 2).

**Figure-14:**
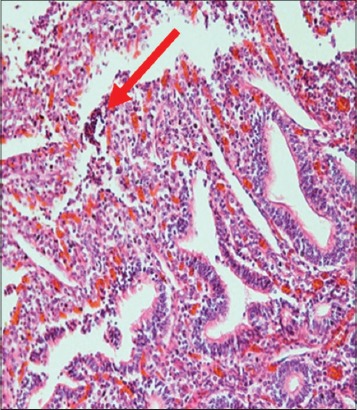
Micrograph of inflammatory cells infiltration in the ileum, H and E, ×200, (Group 2).

**Figure-15:**
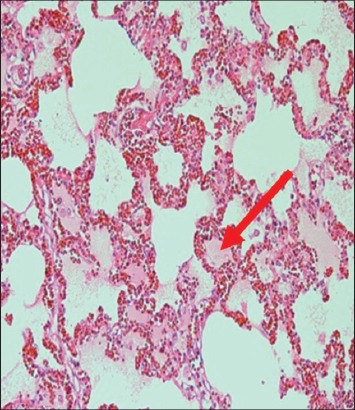
Micrograph of edema in the lung, H and E, ×200, (Group 2).

**Figure 16:**
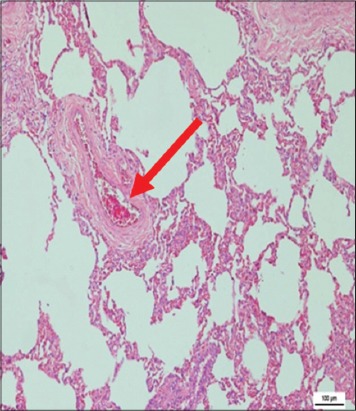
Micrograph of congested and hemorrhagic in the lung, H and E, ×200, (Group 3).

**Figure-17:**
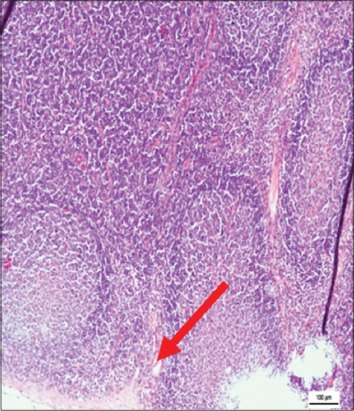
Micrograph of necrosis and degeneration in the submandibular lymph node, H and E, ×200, (Group 3).

**Figure-18:**
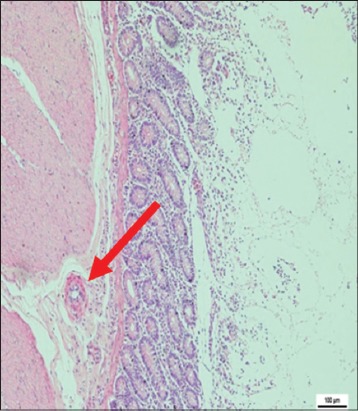
Micrograph of inflammatory cells infiltration in the ileum, H and E, ×200, (Group 3).

**Figure-19:**
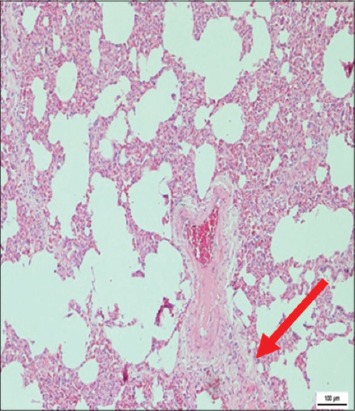
Micrograph of edema in the lung, H and E, ×200, (Group 3).

The immune organs, gastrointestinal tract organs, and the vital organs samples were collected for microscopic examinations. There were no significant histopathology lesions in Group 1 buffaloes. In contrast, buffaloes from Group 2 showed moderate to severe hemorrhage and congestion (Figure-12); necrosis and degeneration (Figure-13); and inflammatory cell infiltration (Figure-14) in all organs. Also, only the lung showed mild to moderate edema lesions (Figure-15). Group 3 buffaloes also showed mild to moderate hemorrhage and congestion (Figure-16); necrosis and degeneration (Figure-17); and inflammatory cell infiltration (Figure-18) in all organs. Similar to Group 2 buffaloes, only the lung showed normal to mild edema lesions in Group 3 buffaloes (Figure-19). There were significant differences (p<0.05) in hemorrhage and congestion; necrosis and degeneration; and inflammatory cells infiltration in organs comparing Groups 1-3 buffaloes. However, there were no significant differences (p>0.05) in edema lesions in all organs except for the lung comparing Group 1 to Group 2 (Table-3) and Group 3 buffaloes (Table-4).

**Table-3:** Histopathological alterations in buffaloes after 12 h of subcutaneous inoculations of *Pasteurella multocida* Type B: 2.

Organs	Hemorrhage and congestion		Necrosis and degeneration		Inflammatory cell infiltration		Edema	
			
	Group 1	Group 2	Group 1	Group 2	Group 1	Group 2	Group 1	Group 2
Bone marrow	0.00±0.19_b_	2.33±0.19_a_	0.00±0.00_b_	1.00±0.00_a_	0.00±0.11_b_	0.83±0.11_a_	0.00±0.00_a_	0.00±0.00_a_
Spleen	0.00±0.18_b_	2.75±0.18_a_	0.00±0.11_b_	2.83±0.11_a_	0.00±0.11_b_	1.83±0.11_a_	0.00±0.00_a_	0.00±0.00_a_
Submandibular lymph node	0.00±0.00_b_	2.00±0.00_a_	0.00±0.14_b_	1.67±0.14_a_	0.00±0.14_b_	1.33±0.14_a_	0.00±0.00_a_	0.00±0.00_a_
Prescapular lymph node	0.00±0.21_b_	1.83±0.21_a_	0.00±0.21_b_	1.83±0.21_a_	0.00±0.11_b_	1.17±0.11_a_	0.00±0.00_a_	0.00±0.00_a_
Femoral lymph node	0.00±0.18_b_	1.75±0.18_a_	0.00±0.11_b_	1.67±0.11_a_	0.00±0.14_b_	0.67±0.14_a_	0.00±0.00_a_	0.00±0.00_a_
Mesenteric lymph node	0.00±0.11_b_	1.83±0.11_a_	0.00±0.11_b_	1.67±0.11_a_	0.00±0.11_b_	0.83±0.11_a_	0.00±0.00_a_	0.00±0.00_a_
Tonsil	0.00±0.14_b_	2.33±0.14_a_	0.00±0.14_b_	1.33±0.14_a_	0.00±0.14_b_	0.33±0.14_a_	0.00±0.00_a_	0.00±0.00_a_
Esophagus	0.00±0.08_b_	1.08±0.08_a_	0.00±0.20_b_	1.83±0.20_a_	0.00±0.15_b_	0.50±0.15_a_	0.00±0.00_a_	0.00±0.00_a_
Rumen	0.00±0.19_b_	1.33±0.19_a_	0.00±0.15_b_	1.50±0.15_a_	0.00±0.11_b_	0.83±0.11_a_	0.00±0.00_a_	0.00±0.00_a_
Reticulum	0.00±0.17_b_	1.00±0.17_a_	0.00±0.21_b_	1.83±0.21_a_	0.00±0.21_b_	0.83±0.21_a_	0.00±0.00_a_	0.00±0.00_a_
Omasum	0.00±0.14_b_	1.33±0.14_a_	0.00±0.14_b_	1.67±0.14_a_	0.00±0.11_b_	1.17±0.11_a_	0.00±0.00_a_	0.00±0.00_a_
Abomasum	0.00±0.15_b_	1.50±0.15_a_	0.00±0.17_b_	2.00±0.17_a_	0.00±0.11_b_	1.17±0.11_a_	0.00±0.00_a_	0.00±0.00_a_
Duodenum	0.00±0.22_b_	1.75±0.22_a_	0.00±0.14_b_	1.33±0.14_a_	0.00±0.00_b_	1.00±0.00_a_	0.00±0.00_a_	0.00±0.00_a_
Jejunum	0.00±0.18_b_	1.75±0.18_a_	0.00±0.21_b_	2.17±0.21_a_	0.00±0.00_b_	1.00±0.00_a_	0.00±0.00_a_	0.00±0.00_a_
Ileum	0.00±0.12_b_	2.00±0.12_a_	0.00±0.14_b_	2.33±0.14_a_	0.00±0.15_b_	1.50±0.00_a_	0.00±0.00_a_	0.00±0.00_a_
Caecum	0.00±0.27_b_	2.17±0.27_a_	0.00±0.11_b_	1.83±0.11_a_	0.00±0.14_b_	1.33±0.14_a_	0.00±0.00_a_	0.00±0.00_a_
Colon	0.00±0.15_b_	1.42±0.15_a_	0.00±0.21_b_	1.83±0.21_a_	0.00±0.11_b_	1.17±0.11_a_	0.00±0.00_a_	0.00±0.00_a_
Rectum	0.00±0.24_b_	2.17±0.24_a_	0.00±0.21_b_	2.17±0.21_a_	0.00±0.14_b_	0.67±0.14_a_	0.00±0.00_a_	0.00±0.00_a_
Trachea	0.00±0.15_b_	0.50±0.15_a_	0.00±0.17_b_	1.00±0.17_a_	0.00±0.14_b_	0.33±0.14_a_	0.00±0.00_a_	0.00±0.00_a_
Lung	0.00±0.00_b_	3.00±0.00_a_	0.00±0.00_b_	3.00±0.00_a_	0.00±0.15_b_	1.50±0.15_a_	0.00±0.25_b_	1.00±0.25_a_
Heart	0.00±0.15_b_	1.58±0.15_a_	0.00±0.22_b_	1.67±0.22_a_	0.00±0.11_b_	1.17±0.11_a_	0.00±0.00_a_	0.00±0.00_a_
Liver	0.00±0.15_b_	2.50±0.15_a_	0.00±0.15_b_	2.50±0.15_a_	0.00±0.00_b_	1.00±0.00_a_	0.00±0.00_a_	0.00±0.00_a_
Kidney	0.00±0.13_b_	2.75±0.13_a_	0.00±0.14_b_	2.67±0.14_a_	0.00±0.15_b_	1.50±0.15_a_	0.00±0.00_a_	0.00±0.00_a_

All values are expressed as mean±SE;

a,b values with superscript within rows are significantly different at p<0.05, SE=Standard error

**Table-4:** Histopathological alterations in buffaloes after 21 days of oral inoculations of *Pasteurella multocida* Type B: 2.

Organs	Hemorrhage and congestion		Necrosis and degeneration		Inflammatory cell infiltration		Edema	
			
	Group 1	Group 3	Group 1	Group 3	Group 1	Group 3	Group 1	Group 3
Bone Marrow	0.00±0.13_a_	0.25±0.13_a_	0.00±0.08_b_	0.92±0.08_a_	0.00±0.08_a_	0.08±0.08_a_	0.00±0.00_a_	0.00±0.00_a_
Spleen	0.00±0.13_a_	0.25±0.13_a_	0.00±0.15_b_	0.92±0.15_a_	0.00±0.00_a_	0.00±0.00_a_	0.00±0.00_a_	0.00±0.00_a_
Submandibular lymph node	0.00±0.19_b_	0.50±0.19_a_	0.00±0.26_b_	1.58±0.26_a_	0.00±0.11_a_	0.17±0.11_a_	0.00±0.00_a_	0.00±0.00_a_
Prescapular lymph node	0.00±0.13_a_	0.25±0.13_a_	0.00±0.28_b_	1.50±0.28_a_	0.00±0.11_a_	0.17±0.11_a_	0.00±0.00_a_	0.00±0.00_a_
Femoral lymph node	0.00±0.15_b_	0.50±0.15_a_	0.00±0.17_b_	1.83±0.17_a_	0.00±0.19_b_	0.50±0.19_a_	0.00±0.00_a_	0.00±0.00_a_
Mesenteric lymph node	0.00±0.18_b_	0.67±0.18_a_	0.00±0.19_b_	1.58±0.19_a_	0.00±0.15_b_	0.50±0.15_a_	0.00±0.00_a_	0.00±0.00_a_
Tonsil	0.00±0.15_b_	0.42±0.15_a_	0.00±0.25_b_	1.25±0.25_a_	0.00±0.15_b_	0.92±0.15_a_	0.00±0.00_a_	0.00±0.00_a_
Esophagus	0.00±0.08_b_	0.92±0.08_a_	0.00±0.15_b_	0.42±0.15_a_	0.00±0.00_a_	0.00±0.00_a_	0.00±0.00_a_	0.00±0.00_a_
Rumen	0.00±0.14_b_	0.67±0.14_a_	0.00±0.15_b_	0.50±0.15_a_	0.00±0.13_a_	0.25±0.13_a_	0.00±0.00_a_	0.00±0.00_a_
Reticulum	0.00±0.15_b_	0.58±0.15_a_	0.00±0.22_b_	0.67±0.22_a_	0.00±0.13_a_	0.25±0.13_a_	0.00±0.00_a_	0.00±0.00_a_
Omasum	0.00±0.15_b_	0.58±0.15_a_	0.00±0.12_b_	1.00±0.12_a_	0.00±0.11_a_	0.17±0.11_a_	0.00±0.00_a_	0.00±0.00_a_
Abomasum	0.00±0.14_b_	0.67±0.14_a_	0.00±0.17_b_	1.00±0.17_a_	0.00±0.15_b_	0.50±0.15_a_	0.00±0.00_a_	0.00±0.00_a_
Duodenum	0.00±0.08_b_	0.92±0.08_a_	0.00±0.18_b_	1.25±0.18_a_	0.00±0.15_b_	0.50±0.15_a_	0.00±0.00_a_	0.00±0.00_a_
Jejunum	0.00±0.11_b_	0.83±0.11_a_	0.00±0.19_b_	0.92±0.19_a_	0.00±0.22_b_	0.67±0.22_a_	0.00±0.00_a_	0.00±0.00_a_
Ileum	0.00±0.00_b_	1.00±0.00_a_	0.00±0.21_b_	1.00±0.21_a_	0.00±0.11_b_	0.83±0.11_a_	0.00±0.00_a_	0.00±0.00_a_
Caecum	0.00±0.13_b_	0.75±0.13_a_	0.00±0.19_b_	0.67±0.19_a_	0.00±0.13_b_	0.75±0.13_a_	0.00±0.00_a_	0.00±0.00_a_
Colon	0.00±0.08_a_	0.08±0.08_a_	0.00±0.15_b_	0.92±0.15_a_	0.00±0.13_b_	0.75±0.13_a_	0.00±0.00_a_	0.00±0.00_a_
Rectum	0.00±0.15_b_	0.58±0.15_a_	0.00±0.18_b_	0.75±0.18_a_	0.00±0.08_b_	0.92±0.08_a_	0.00±0.00_a_	0.00±0.00_a_
Trachea	0.00±0.14_b_	0.67±0.14_a_	0.00±0.15_b_	0.92±0.15_a_	0.00±0.08_a_	0.08±0.08_a_	0.00±0.00_a_	0.00±0.00_a_
Lung	0.00±0.17_b_	2.17±0.17_a_	0.00±0.15_b_	2.50±0.15_a_	0.00±0.17_b_	1.17±0.17_a_	0.00±0.20_b_	0.89±0.20_a_
Heart	0.00±0.15_b_	1.58±0.15_a_	0.00±0.12_b_	2.00±0.12_a_	0.00±0.08_b_	0.92±0.08_a_	0.00±0.00_a_	0.00±0.00_a_
Liver	0.00±0.13_b_	1.25±0.13_a_	0.00±0.14_b_	1.33±0.14_a_	0.00±0.13_b_	1.25±0.13_a_	0.00±0.00_a_	0.00±0.00_a_
Kidney	0.00±0.19_b_	1.42±0.19_a_	0.00±0.19_b_	1.08±0.19_a_	0.00±0.13_b_	0.75±0.13_a_	0.00±0.00_a_	0.00±0.00_a_

All values are expressed as mean±SE;

a,b values with superscript within rows are significantly different at p<0.05, SE=Standard error

## Discussion

In most cases, the clinical findings of HS are either acute or peracute, resulting in death within 8-24 h after onset [11]. The disease is more susceptible in young animals ranging from 6 months to a year old [1,16]. Infected animals may be found with elevated temperature, submandibular edema, congested mucous membrane and respiratory distress with profuse nasal discharged [1,3,7]. In this study, all buffaloes were 8 months old where Groups 2 and 3 buffaloes showed typical HS signs such as pyrexia, submandibular edema, congested mucous membrane, nasal discharges and labored breathing. Group 3 buffaloes only showed mild clinical signs such as elevated temperature and serous nasal discharge. The differences in clinical signs can be explained through the route of inoculation. Experimentally, subcutaneous inoculation results in rapid onset and produced more consistent results compared to intranasal or oral route [2,18]. This was also supported by an experiment where orally inoculated buffaloes revealed milder clinical signs as compared to intra-tracheally infected buffaloes [14,15]. The subcutaneous group was only able to survive for 12 h before euthanasia due to recumbency and respiratory distress. However, the oral group was able to survive throughout the 21 days of the experiment.

Clinical pathology such as hematology and biochemistry is of great help to the clinician in arriving at a correct diagnosis, prognosis and efficacy of a treatment [19]. During a bacterial infection, hematological and biochemistry changes are first detected during routine blood sampling. However, animal’s defense mechanism can react quite differently, and there is no singular pattern in complete blood count that indicates a bacterial infection [20]. There were some hematological and biochemical markers that can be used for early detection in animals infected with wild Type of *P. multocida* [21,22]. Nevertheless, the blood results obtained comparing the subcutaneous and oral group infected buffaloes were documented for the first time. Buffaloes infected subcutaneously were having erythrocytosis and leukopenia, however; buffaloes infected orally were having leukocytosis throughout the experiment. It is common for cattle with acute bacterial infections such as the subcutaneous group to have neutropenia because of the small storage pool of segmented neutrophils in the bone marrow [22]. Severe inflammation due to the bacteria endotoxin also contributes to neutropenia due to neutrophil migration and emigration into inflamed tissue exceed the release of neutrophil from the bone marrow [23]. However, within days, neutrophil production and release may result in neutrophilia, which was observed in the oral group. On the other hand, the result of this study revealed significant increase in red blood cells in the subcutaneous group. This is not consistent with findings who concluded that inflammation is able to reduce red blood count leading to anemia [24]. This can be due to dehydration and shock that occurred in buffaloes infected subcutaneously as HS is an acute and hemorrhagic disease.

Histopathology focuses on the interrelationship and integration of molecular and physiological activities within the body [25]. The earliest report on the histopathological changes in HS was done experimentally in bison calf using B2 strain. In the present study, subcutaneous and oral group buffaloes had different post mortem and histopathology findings [17]. Grossly subcutaneous group showed generalized congestion, hyperemia and hemorrhage in the vital organs, gastrointestinal organs, and immune organs. These findings were consistent with previous HS report and experimental findings [1,2,7,16,17,19,26-29]. In contrast, orally infected group only had mild lesions in the lung and liver. These findings were supported by previous experiments, who stated that buffalo calves inoculated orally showed milder lesions compared to other route of infections [2,14,15]. The lesions such as hemorrhage, edema, and white blood cells infiltration were observed in the lung, lymph nodes, spleen, gastro-intestinal tract, liver, kidney and the heart [27]. Nevertheless, in our study, orally infected group was showing milder histopathological lesions compared to the subcutaneous group. Histological lesions of orally infected group were milder compared to other infected group, which is not a typical sign in HS infected animals [14,15]. Oral route may not play a major role in the development of HS but they carried *P. multocida* organism in the gastrointestinal organs, which may act as carrier animal [14,15].

In summary, this study compared the clinical responses, hematology and biochemistry alterations, post mortem changes, and cellular changes in tissues of buffaloes challenged with *P. multocida* Type B:2 via subcutaneous and oral routes. There were no studies were reported previously to observe the differences in buffaloes response using these two routes of infections. In this study, both treatment groups showed significant clinical responses. The subcutaneous group showed severe clinical signs and were in agreement with previous studies [14,15,28,29] and all the buffaloes in this group were euthanized within 7 days of post-infection. The novelty of this study was the oral group where the buffaloes in this treatment group survived although these buffaloes exhibit mild clinical signs. The buffaloes in this group survived throughout the stipulated experimental period of 21 days. The data on hematology and biochemistry responses in these two different groups were documented for the first time and there were no previous literatures on these responses. Moreover, the cellular changes in immune organs of subcutaneous and oral groups were added knowledge in HS studies in buffaloes. From this study, we may conclude that oral route infection of *P. multocida* Type B:2 in buffaloes may stimulate the host cell responses. More studies is needed if there is possibilities of oral vaccine through feed that may be developed in future where vaccine administration via feed may increase the coverage of vaccination percentage although oil adjuvant vaccines have been used routinely to control HS, outbreaks among vaccinated animals are not uncommon due to the difficulty of vaccine administration [4,30].

## Conclusions

There were changes in clinical signs, blood parameters, post mortem and histopathology following experimental-infection with *P. multocida* Type B:2 via oral route of exposure. This route of infection could lead to mild clinical responses, alteration in hematology and biochemistry, gross lesions in the lung and liver with mild to moderate histopathology modifications.

## Authors’ Contributions

FFJA, MZS, AWH, AAS, MAML, ARO, and MZAB conceptualized and supervised the research. ELTC, LA, ADM, HHI and MJN collected samples, drafted and revised the manuscript and done statistical analysis. All authors have read and approved the manuscript.
